# Certification of New Selenium-Enriched Yeast and Supplement Reference Materials for Selenomethionine Using Two Independent Measurement Strategies

**DOI:** 10.3390/molecules29010235

**Published:** 2024-01-01

**Authors:** Xiao Li, Ling Shi, Panshu Song, Wei Cai, Ximing Luo, Bo Zhao

**Affiliations:** 1 School of Ocean Sciences, China University of Geosciences (Beijing), Beijing 100083, China; lixiao@nim.ac.cn; 2 Division of Chemical Metrology and Analytical Science, National Institute of Metrology, Beijing 100029, China; shiling@nim.ac.cn (L.S.); songpsh@nim.ac.cn (P.S.); caiwei@nim.ac.cn (W.C.)

**Keywords:** selenium speciation, selenomethionine, certified reference material, HPLC-ICP-MS, HPLC-ESI-MS/MS

## Abstract

Selenium-enriched yeast possesses the unique ability of transforming chemical selenium, such as sodium selenite, into a biologically active form, which mitigates its toxic effects on the human body. The transformation product of this process, selenomethionine, can be safely and effectively absorbed and utilized by the human body; hence, it has been spiked into a selenium-enriched supplement. This study employs two distinct measurement strategies to determine the selenomethionine content in two candidate reference materials, a selenium-enriched yeast powder and supplement, using both organic and inorganic mass spectrometry. The concentrations of selenomethionine in the selenium-enriched yeast were determined using HPLC-ICP-MS and HPLC- ESI-MS/MS, with mass fractions measured at 718 mg SeMet kg^−1^ and 715 mg SeMet kg^−1^, respectively. Notably, both methods yielded consistent results for the selenium supplement, with a selenomethionine mass fraction of 59 mg SeMet kg^−1^. Ultimately, the certified values of these candidate reference materials were determined as 716 mg kg^−1^ and 59 mg SeMet kg^−1^ with expanded uncertainties of 36 mg SeMet kg^−1^ (*k* = 2) and 5 mg SeMet kg^−1^ (*k* = 2), respectively. The development of these candidate reference materials serves as a valuable reference for diverse methods aiming to determine the value of organic selenium speciation in complex food substrates.

## 1. Introduction

Selenium was first discovered and named by the Swedish chemist Jöns Jacob Berzelius in 1817. It was not until 1973 that the World Health Organization officially recognized selenium as an essential trace element for the human body [1]. Numerous studies have shown that selenium has a variety of beneficial functions, including improving immunity, exhibiting anti-oxidation and anti-tumor properties, and protecting cells [2]. The maximum safe dietary selenium intake is suggested as 400 mg per day. A level of ∼40 mg daily is suggested as the minimum amount required and an intake of <11 m g daily will definitely put a person at risk of deficiency problems [3]. China is a typical country with selenium deficiency, and the distribution of selenium on the Earth’s surface is uneven. In areas with serious selenium deficiency, such as Northeast China, the low content of selenium in food has led to endemic diseases, such as Keshan disease and Kaschin–Beck disease [4]. Selenium in food can be divided into inorganic selenium and organic selenium. The utilization rate of organic selenium in animal bodies is much higher than that of inorganic selenium [5]. An insufficient or excessive intake of selenium may cause adverse effects on the human body [6]. Natural foods, such as selenium-rich plants, are an effective way to increase selenium intake, while they may not be enough to meet the recommended daily intake for the human body [7]. Therefore, studies on understanding the enrichment process of selenium in plants, animals, and microorganisms and comparing the composition of natural selenium-rich foods and artificially transformed selenium-rich foods have been carried out to find suitable selenium-rich products to supplement the human body’s demand for selenium [8].

Natural selenium-rich foods are primarily derived from organic selenium-rich raw materials formed by the biotransformation of crops in inorganic-selenium-rich soils [9]. In general, natural selenium-rich foods include selenium-rich tea [10], selenium-rich rice [11], selenium-rich garlic [12], and other similar products. Artificial conversion, on the other hand, involves using plants, animals, and microorganisms as transformants to convert inorganic selenium into organic selenium [13], which can then be processed and used to produce selenium-rich foods, such as selenium-rich eggs [14] and selenium-rich pork [15]. As far as we know, the content and biological activity of selenium in natural selenium-containing compounds found in plants and animals are relatively lower than those of selenium in the synthesized selenium-containing compounds [16]. Selenium-enriched yeast, as a source of organic selenium, has a higher bioavailability and better safety compared to inorganic selenium, and is an ideal selenium supplement with broad application prospects. It can be used as a nutrient-rich additive in health-food products and as a starter to prepare selenium-rich food [17]. SeMet is the main component of organic selenium in selenium-rich yeast that accounts for more than half of the organic selenium content [18]. Selenium supplements contain selenium in different chemical forms. The current evidence favors SeMet over the other forms of selenium. Therefore, in the majority of supplements, selenium is present as SeMet [19].

At present, chromatographic separation methods, such as gas chromatography (GC) [20], high-performance liquid chromatography (HPLC) [21], and capillary electrophoresis (CE) [22], are commonly employed to detect SeMet in food owing to their advantages of high selectivity and sensitivity. Among these methods, liquid chromatography is the most important separation method, which is often coupled with various detection techniques, such as high-performance liquid chromatography-inductively coupled plasma mass spectrometry (HPLC-ICP-MS) [21], high-performance liquid chromatography-atomic fluorescence spectroscopy (HPLC-AFS) [23], and HPLC-fluorescence detection [24].

The compliance with respect to the quality can be very well assured by the use of certified reference materials (CRMs) [25]. In this paper, to ensure the accurate and reliable quantification of SeMet in selenium-enriched nutritional supplements, the first certified reference materials for SeMet based on a selenium-enriched yeast and supplement are developed. The selenium-enriched yeast and supplement were purchased from the market, and the candidate material of the CRMs were obtained through homogenization and sieving. The certified value of the CRMs were determined by HPLC-ICP-MS/MS and HPLC-ESI-MS/MS approaches. The homogeneity and stability under storage and transportation conditions of the CRM were monitored, and their associated uncertainties were taken into account in the total uncertainty. The development of the two mentioned substances’ standard matrices address the gap in the research of relevant CRMs for food in China. The uncertainty levels of these substances are on par with internationally established CRM levels.

## 2. Results and Discussion

### 2.1. Optimization of the Extraction Method

#### 2.1.1. Enzyme Screening

As demonstrated by Ma et al. [26], the use of a combination of protease XIV with other enzymes not only failed to increase the extraction efficiency, but also enhanced the blank levels of Se species. Thus, only protease XIV was utilized for SeMet extraction in the following studies.

#### 2.1.2. Optimization of the Solvent for the Enzyme Solution Based on Enzymatic Hydrolysis

This study demonstrated that overnight enzymatic hydrolysis yields a SeMet recovery rate of less than 50%. To enhance the stability of the target compound, the laboratory utilized 0.1 M Tris-HCl, 0.1% trifluoroacetic acid (TFA), 5 mmol/L citric acid, and ultrapure water as the solvents for enzyme preparation. The results reveal that, when 0.1 M Tris-HCl was employed as the solvent, the spike recovery of SeMet was 102.3%, markedly higher than the recovery rates obtained with the other three solvents (0.1% TFA: 67.1%; 5 mmol/L citric acid: 73.9%; and ultrapure water: 44.7%). Consequently, 0.1 M Tris-HCl was selected as the solvent for enzyme preparation in the experiment.

#### 2.1.3. Ultrasound-Assisted Enzymatic Hydrolysis

To mitigate the potential contamination issues stemming from Tris-HCl of the ion source of HPLC-ESI-MS/MS, the laboratory devised an ultrasound-assisted enzymatic hydrolysis method for extracting SeMet from selenium-enriched yeast. This involved weighing 0.2 g of the sample and adding 5 g of the protease XIV enzyme solution (10 mg mL^−1^). The mixture was then sonicated at 37 °C for 1 h. Following centrifugation at 8000 rpm for 10 min, the supernatant was extracted. Subsequently, another 5 g of the enzyme solution was added to the centrifuge tube, and the extraction process was repeated. The supernatants from both centrifugations were combined and filtered through a 0.2 μm polyethersulfone (PES) membrane and used for analysis. The results demonstrate that, with this pretreatment method, the recovery of the target compound reached 97.9%.

### 2.2. Optimization of the Mobile Phase and Chromatographic Column

**Figure 1:**
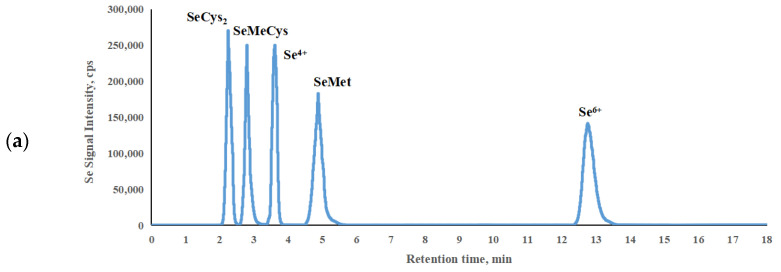

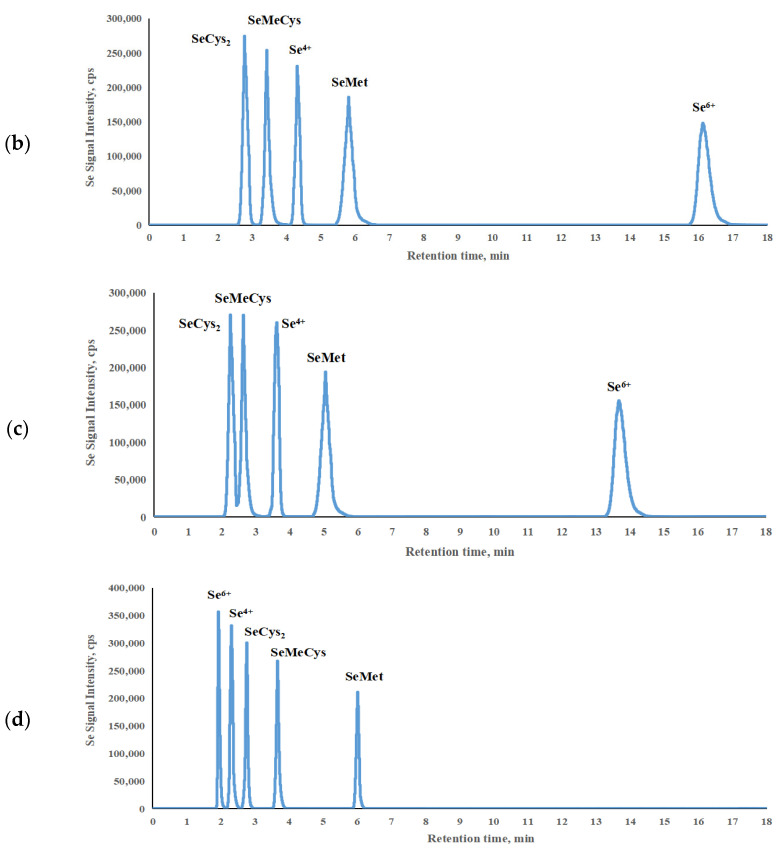
The chromatographic separation results of the different mobile phases and chromatographic column. (**a**) 40 mmol/L diammonium hydrogen phosphate with the anion exchange column; (**b**) 5 mmol/L citric acid with the anion exchange column; (**c**) 60 mmol/L ammonium dihydrogen phosphate with the anion exchange column and (**d**) A total of 10 mmol/L sodium 1-butanesulfonic acid, 8 mmol/L tetramethylammonium hydroxide pentahydrate, and 5 mmol/L malonic acid with the C_18_ column.

Due to the structural and property similarities between selenium and arsenic species, this experiment initially attempted to utilize a PRP-X100 anion exchange column as the chromatographic column. Diammonium hydrogen phosphate (Figure 1a), an ideal mobile phase for arsenic speciation analysis, was compared with two previously reported mobile phases (citric acid [6] (Figure 1b) and ammonium dihydrogen phosphate [27] (Figure 1c)). Through the optimization of the concentration, pH, and flow rate of different mobile phases, the results demonstrate that the chromatographic separation and analysis time were optimal when using diammonium hydrogen phosphate as the mobile phase.

Considering that selenate, as a commonly occurring selenium species in the samples, tends to have a prolonged retention time, which significantly affects the analysis efficiency, our laboratory explored the use of a C_18_ reversed-phase column. This approach involves the rapid elution of inorganic selenium, effectively reducing the analysis time for selenium species. The use of an OSAKA SODA CAPCELL PAK C_18_ reversed-phase column with a mobile phase consisting of 10 mmol/L sodium 1-butanesulfonic acid, 8 mmol/L tetramethylammonium hydroxide pentahydrate, 5 mmol/L malonic acid, and 5% MeOH at a pH of 4.0 exhibited the shortest analysis time and superior separation efficiency. Therefore, these chromatographic conditions were selected for the separation of the selenium species in this study (Figure 1d).

### 2.3. Reproducibility, Linear Relationship, and Detection Limits

The instrument conditions of HPLC-ICP-MS were optimized through instrument tuning. After that, a series of SeMet standard solutions (0.5, 5, 25, 50, and 100 ng g^−1^) were analyzed for linearity assessment. Within the concentration range of 0.5–100 ng/g, the linear correlation coefficient between the peak area of SeMet and its concentration was calculated to be 0.9995. The instrument’s LOD was determined as 0.23 ng g^−1^ based on the calculation of 3 times of signal-to-noise ratio (SNR), and the LOQ of the instrument was established as 0.71 ng g^−1^ based on 10 times of SNR. The precision of the developed method, evaluated using a 25 ng g^−1^ SeMet standard solution, demonstrated a relative standard deviation (RSD) of 0.52% (*n* = 6).

In the HPLC-ESI-MS/MS analysis, a highly favorable linear correlation coefficient of 0.9998 was achieved for a series of standard solutions with the same concentrations mentioned above. The instrument’s LOD was found to be 0.5 ng g^−1^, while the LOQ was determined to be 1.7 ng g^−1^. In addition, at a SeMet standard solution concentration of 25 ng g^−1^, the precision, as indicated by the relative standard deviation (RSD) of the method, was exceptionally low at 1.2% (*n* = 6). This demonstrates the method’s high precision and reliability in quantifying SeMet concentrations.

### 2.4. Homogeneity Test

The homogeneity of the candidate certified reference materials (CRMs) for SeMet was evaluated using the HPLC-ICP-MS method. Homogeneity testing was conducted on 11 units for each of the two CRM substance candidates. For each unit, two parallel samples were taken and subjected to analysis. The calculated *F* values for SeMet (*F*_calculated-yeast_ = 1.59 and *F*_calculated-supplement_ = 1.16) were found to be lower than the critical value of *F*_0.05(10, 11)_ = 2.85, indicating that no significant differences were evident within and between the sample packets. Thus, it can be concluded that the homogeneity for the CRM candidates was satisfied. The uncertainty introduced by heterogeneity (*u_bb_*) was assessed based on the inhomogeneity among the bottles (*M_among_*) and within the bottles (*M_within_*). The *u_bb_* was calculated as shown in Equation (1):

(1) $$u_{bb}=\sqrt{(M_{among}-M_{within})/n}$$

### 2.5. Stability Monitoring

To assess the stability of the candidate reference materials, both long-term and short-term stability outcomes were evaluated under storage conditions of −20 °C for 12 months and transport temperatures of 50 °C for 5 days. The stability was assessed through a regression line analysis. The t-test results indicate that the SeMet content in both CRMs displayed no statistically significant instability, neither within the elapsed time of 12 months nor under extreme temperatures. The uncertainties for both long-term and short-term stability outcomes were calculated using the following Equation (2).

(2) $$u_{lts}=s(\beta_{1})\times t_{l};\;u_{sts}=s(\beta_{1})\times t_{s}$$

where *s*(*β*_1_) is the standard deviation (SD) of the slope *β*_1_ and t represents the monitoring time of long-term and short-term stability outcomes.

### 2.6. Value Assignment

The certified value of SeMet concentration was obtained through two independent measurement methods, HPLC-ICP-MS and ESI-HPLC-MS/MS. The concentrations of selenomethionine in the selenium-enriched yeast were determined as 718 mg SeMet kg^−1^ and 715 mg SeMet kg^−1^, respectively, while the selenomethionine mass fractions in the selenium supplement were determined as 59 mg SeMet kg^−1^ by both methods. Statistical tests, including Dixon’s Q test and Cochran’s test, were performed, and no data were excluded. Due to the equal accuracy of the results obtained from both measurement methods, the certified values of the selenium-enriched yeast powder and supplement were established as 716 mg/kg and 59 mg/kg, respectively, which represent the average values of the two measurement results.

### 2.7. Uncertainty Assessment

The uncertainty outcomes of the certified SeMet value in the selenium-enriched yeast and supplement were evaluated in accordance with the ISO Guide 35 “Reference materials-Guidance for characterization and assessment of homogeneity and stability”, and were based on the combination of uncertainties associated with characterization ($u_{char}$), possible variations between packets ($u_{bb})$, long-term stability ($u_{lts})$, and short-term stability ($u_{sts})$.

The uncertainties related to the standard solution, sample preparation, and measurement repeatability were all taken into account in the characterization uncertainty. Among them, the uncertainty of preparing the standard solution (*u_std_*) included three components: *u_CRM_* for the uncertainty of the CRMs for SeMet, *u_mCRM_* for the precision of weighing the CRMs, and *u_mCRMsol_* for the precision of weighing the solvent. The formula for *u_std_* is as shown in Equation (3):

(3) $$u_{std}=\sqrt{u_{CRM}^{2}+u_{mCRM}^{2}+u_{mCRMsol}^{2}}$$

The uncertainty of preparing the sample *u_sam_* included *u_msam_* and *u_msamsol_*. Here, *u_msam_* represents the uncertainty from the weighing of the sample and *u_msamsol_* represents the uncertainty from the weighing of the solvent for the sample. The formula for *u_sam_* is as shown in Equation (4):

(4) $$u_{sam}=\sqrt{u_{msam}^{2}+u_{msamsol}^{2}}$$

The uncertainty of the measurement results according to the instrument stability and sample preparation procedures from the measurement repeatability for HPLC-ICP-MS (*u_AICP_*) and ESI-HPLC-MS/MS (*u_AESI_*) are calculated using the following Equations (5) and (6), respectively:

(5) $$u_{AICP}=\sqrt{{\sum_{i=1}^{n}\left(x-\bar{x}\right)}^{2}/n(n-1)}$$

(6) $$u_{AESI}=\sqrt{{\sum_{i=1}^{n}\left(x-\bar{x}\right)}^{2}/n(n-1)}$$

where *x* is the result of the subsamples, $\bar{x}$ is the average test result of all subsamples, and *n* is the number of measurement repetitions.

The combined uncertainty outcomes of the definite values of HPLC-ICP-MS *u_charICP_* and ESI-HPLC-MS/MS *u_charESI_* are calculated using *u_AICP_*, *u_AESI_*, *u_std_*, and *u_sam_*, as shown in Equations (7) and (8), respectively:

(7) $$u_{charICP}=\sqrt{u_{std}^{2}+u_{sam}^{2}+u_{AICP}^{2}}$$

(8) $$u_{charESI}=\sqrt{u_{std}^{2}+u_{sam}^{2}+u_{AESI}^{2}}$$

As the results obtained from two independent measurement methods reveal equal precision values, the combined relative uncertainty of the definite value of *u_char_* is calculated using *u_charICP_* and *u_charESI_*, as shown in Equation (9):

(9) $$u_{char}=\sqrt{u_{charICP}^{2}+u_{charESI}^{2}}$$

The combined uncertainty of the certified *u_C_* is calculated as shown in Equation (10):

(10) $$u_{C}=\sqrt{u_{char}^{2}+u_{bb}^{2}+u_{lts}^{2}+u_{sts}^{2}}$$

Finally, the expanded uncertainty *U_CRM_* values were calculated as 36 mg/kg and 5 mg/kg according to the following Equation (11):

(11) $$U_{CRM}=u_{C}\times k(k=2)$$

The uncertainty values of SeMet in the selenium-enriched yeast powder and supplement CRMs are shown in Table 1 and Table 2, respectively. Ultimately, the expanded uncertainties of these candidate reference materials were calculated as 36 mg SeMet kg^−1^ (*k* = 2) and 5 mg SeMet kg^−1^ (*k* = 2), respectively, which were as low as the uncertainties of the selenomethionine CRMs produced by other National Metrology Institutes.

## 3. Materials and Methods

### 3.1. Candidate Reference Materials

The selenium-enriched yeast powder samples were purchased from a commercial supplier. The raw material for the selenium-enriched supplement was obtained through the preliminary screening of various commercially supplements. It was confirmed that the composition included organic selenium in the form of SeMet. Some products are falsely advertised as containing only inorganic selenium components. Therefore, they cannot be considered as raw materials. To ensure homogeneity, the samples were sieved using a 100-mesh sieve and then thoroughly mixed for 1 h in a V-type aluminum alloy mixer. The resulting mixture was divided into 200 packets, each containing 5 g of the selenium-enriched yeast powder or supplement, and sealed within aluminum bags. To prevent contamination by pathogenic or spoilage microorganisms, the candidates of the standard material were sterilized under ^60^Co exposure, delivering an absorbed dose of approximately 2.5 kGy. The irradiated samples were subsequently stored at a temperature of −20 °C to ensure long-term preservation.

### 3.2. Materials and Apparatus

The CRM of selenomethionine in water, GBW10034, was developed by the National Institute of Metrology (Beijing, China) and has a certified value of (39.4 ± 1.0) μg g^−1^ as Se. A selenium-enriched yeast CRM, SELM-1, which was purchased from the National Research Council Canada (NRC, Ottawa, Canada), was used for the method validation. The certified value of SeMet was (3190 ± 260) mg kg^−1^. Protease XIV from Sigma-Aldrich Inc. (St. Louis, MO, USA) was employed for the extraction of SeMet from the yeast sample. HPLC-grade methanol, obtained from Thermo Fisher Scientific (Carlsbad, CA, USA), was used as the organic solvent. Ultrapure water was produced using a Milli-Q Integral 5 system (Millipore Corp., Burlington, MA, USA) and was utilized for the preparation of all samples and standard solutions. For the mobile phase preparation, HPLC-grade 1-butanesulfonic acid sodium salt, malonic acid (Regent Plus, 99%), and tetramethylammonium hydroxide pentahydrate (HPLC-grade) were supplied by Merck (Darmstadt, Germany).

The ultrasound-assisted extractions were conducted using an ultrasonic bath (KQ-500GDV, Kun Shan Ultrasonic Instruments Co., Ltd., Kunshan, Jiangsu, China). Shaking was performed using a WS20 shaking incubator (Wiggens, Straubenhardt, Germany). The extracts obtained from the samples were centrifuged using a Universal 320R centrifuge (Hettich, Tuttlingen, Germany). Se determination was carried out using an ICP-MS apparatus (8800, Agilent Technologies, Santa Clara, CA, USA) equipped with a collision cell, a Scott double-pass spray chamber, and a PFA nebulizer. The Agilent 1290 HPLC system (Santa Clara, CA, USA) outlet was directly connected to the MicroMist nebulizer of the ICP-MS/MS via PEEK capillary tubing for SeMet determination. Liquid chromatography tandem with triplequad 3500 mass spectrometer was also employed for the value assignment of SeMet in the yeast powder and supplement.

### 3.3. Preparation of the Standard Solutions

The stock standard solution was prepared by the gravimetric weighing of a certain amount of GBW10034 in ultrapure water to achieve a concentration of 1 mg kg^−1^. It was then sealed in ampoules and stored at a temperature of 4 °C.

### 3.4. Selenomethionine Extraction

To characterize the content values of SeMet in the yeast powder and supplement using (HPLC-ICP-MS and ESI-HPLC-MS/MS), two different extraction methods were developed.

#### 3.4.1. Extraction Method for the HPLC-ICP-MS Analysis

For the HPLC-ICP-MS analysis, 0.2 g of the sample was placed into a 15 mL polypropylene centrifuge tube. Subsequently, 15 mg of protease XIV in 2 mL of 0.1 M Tris-HCl buffer adjusted to a pH of 6.5 was added. The mixture was vortexed for 1 minute and shaken at 180 rpm for 20 h at 37 °C, with vigorous shaking every 60 min. Following this, the mixture was centrifuged at 8000 rpm for 10 min and then filtered through a 0.22 μm PES filter before the HPLC-ICP-MS/MS analysis.

#### 3.4.2. Extraction Method for the ESI-HPLC-MS/MS Analysis

For the ESI-HPLC-MS/MS analysis, an enzymatic hydrolysis method assisted by ultrasound was employed to ensure the stability of SeMet. Therefore, 0.2 g of sample was introduced into a 15 mL polypropylene centrifuge tube. Subsequently, 20 mg of protease XIV was added to 2 mL of ultrapure water. The mixture underwent vortexing for 1 minute, followed by ultrasound treatment for 1 h in a 37 °C water bath, with vigorous shaking every 20 min. After this, the mixture underwent centrifugation at 8000 rpm for 10 min. The extracts were then filtered through a 0.22 μm PES filter before the ESI-HPLC-MS/MS analysis. It is noteworthy that the Tris-HCl buffer was deliberately excluded from this method to prevent the potential contamination of the ESI.

### 3.5. Determination of SeMet by HPLC-ICP-MS

For the analysis of SeMet using HPLC-ICP-MS, the outlet of the HPLC column was directly connected to the MicroMist nebulizer of the ICP-MS/MS instrument (Agilent 8800, Tokyo, Japan) using a PEEK capillary tube. To guarantee precise measurements, the instrument operating conditions were optimized using a mixed solution containing Li, Y, Ce, Tl, and Co elements at a concentration of 1 ng g^−1^.

The chromatographic separation of SeMet was conducted using an Agilent 1290 HPLC system (Santa Clara, CA, USA). A stationary phase consisting of an OSAKA SODA CAPCELL PAK C_18_ column (4.6 × 250 mm, particle size = 5 μm) sourced from Chinese Taipei, China, was utilized. The guard column of the same stationary phase was positioned before the separation columns. Before use, a column was preconditioned following the manufacturer’s instructions. The pH value of the mobile phase in the HPLC was adjusted using a Mettler Toledo FiveEasy Plus pH meter (Zurich, Switzerland). Chromatographic data were collected, stored, and processed using the Agilent software MassHunter (version B.01.03). Even though no significant memory effect was detected, monitoring was conducted by periodically measuring a blank solution to ensure the absence of any memory effect or cross-contamination during the analysis. Table 3 summarizes the typical operating parameters of the ICP-MS system.

### 3.6. Determination of SeMet by ESI-HPLC-MS/MS

**Figure 2:**
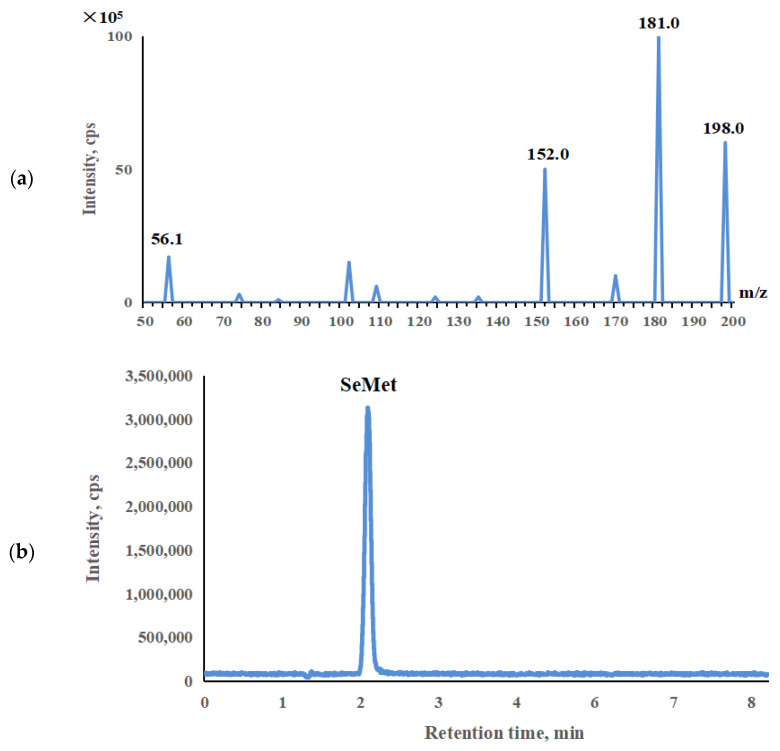
(**a**) MS/MS spectrum of SeMet; (**b**) ESI-HPLC-MS/MS for SeMet in the selenium-enriched yeast powder samples.

The chromatographic column employed for the ESI-HPLC-MS/MS system was a CAPCELL CORE C_18_ column with the dimensions of 3.0 × 100 mm and a particle size of 2.7 μm, manufactured in Chinese Taipei. The mobile phase was operated in the isocratic mode, which consisted of 2 mmol L^−1^ ammonium formate and 5% methanol. The flow rate was 0.25 mL min^−1^. The column temperature was maintained at 30 °C, and a sample injection volume of 2 μL was used. The mass spectrometry analysis was carried out in the positive ion mode with a capillary voltage set at 1.0 kV and a temperature of 550 °C. The selected SeMet ions were monitored using the multiple reaction monitoring (MRM) mode, specifically at m/z 198.0 → 181.0 and 198.0 → 152.0 for SeMet (Figure 2a).

### 3.7. Homogeneity Test

To assess the homogeneity of the SeMet content in the yeast powder and supplement, 11 packets of the samples were randomly selected. Within each packet, two subsamples were subjected to analysis using the HPLC-ICP-MS method to determine the SeMet concentration. The obtained data were subjected to a one-way analysis of variance (ANOVA) at a 95% confidence level. The homogeneity of the property value of the yeast powder and supplement CRMs were evaluated using the *F*-test.

### 3.8. Stability Test

The stability outcomes of the SeMet content in the yeast powder and supplement were assessed by analyzing samples stored at −20 °C for 12 months and subjecting them to conditions simulating possible transportation (at 50 °C) for 0, 1, 2, and 5 days, respectively. For each specified time, three samples were chosen and analyzed using HPLC-ICP-MS. The significance of the observed slope (*β*_1_) was tested using a t-test at a confidence level of 95%.

### 3.9. Value Assignment

According to ISO Guide 35, the value assignment for SeMet was conducted using two distinct analytical methods founded on different principles. For characterization purposes, 9 subsamples were extracted from 3 bottles and analyzed using both HPLC-ICP-MS and ESI-HPLC-MS/MS methods. The obtained measurement results were subjected to the Dixon’s and Cochran’s tests in accordance with the JJF 1343–2022 General and Statistical Principles for the Characterization of Reference Materials.

## 4. Conclusions

In this paper, we successfully developed two novel certified reference materials for SeMet from selenium-enriched yeast powder and supplement. The investigation initially focused on optimizing and comparing various chromatographic conditions for HPLC-ICP-MS. Additionally, for ESI-HPLC-MS/MS, an innovative approach of rapid release of SeMet from the matrix was achieved by implementing an ultrasound-assisted enzymatic hydrolysis method, which ensured the stability of the target analyte while mitigating the potential contamination of the liquid chromatography-mass spectrometry ion source by the extraction solvent. Consequently, the accuracy of the CRMs’ property values was ensured by the integration of both organic and inorganic mass spectrometry techniques in this study.

The certified values of SeMet in the yeast powder and supplement were 716 mg SeMet kg^−1^ and 59 mg SeMet kg^−1^, with expanded uncertainty outcomes of 36 mg SeMet kg^−1^ (*k* = 2) and 5 mg SeMet kg^−1^ (*k* = 2), respectively. These uncertainties revealed the state of the art of the measurement capabilities of SeMet in the matrices of the CRMs. Meanwhile, the results indicate that the SeMet contents in our yeast powder and supplement CRMs are closely aligned with those found in real samples. Therefore, these CRMs will serve as important quality controls for the analysis of SeMet in a wide variety of Se nutritional supplements.

## Figures and Tables

**Table 1:** Uncertainty values of SeMet in the selenium-enriched yeast powder CRM.

Source of Uncertainty	Uncertainty Item	Typical Value (mg/kg)
Homogeneity test	*u_bb_*	9
Long-term stability study	*u_lts_*	13
Short-term stability study	*u_sts_*	8
Standard deviation of the mean values measured by HPLC-ICP-MS	*u_AICP_*	4.15
Standard deviation of the mean values measured by ESI-HPLC-MS/MS	*u_AESI_*	7.88
Standard solution preparation	*u_std_*	9.31
Sample preparation	*u_sam_*	0.68
Relative combined uncertainty	*u_c_*	18
*U_CRM_*	*U_CRM_*	36

**Table 2:** Uncertainty values of SeMet in the selenium-enriched supplement CRM.

Source of Uncertainty	Uncertainty Item	Typical Value (mg/kg)
Homogeneity test	*u_bb_*	1
Long-term stability study	*u_lts_*	1
Short-term stability study	*u_sts_*	1
Standard deviation of the mean values measured by HPLC-ICP-MS	*u_AICP_*	1.27
Standard deviation of the mean values measured by ESI-HPLC-MS/MS	*u_AESI_*	1.75
Standard solution preparation	*u_std_*	0.77
Sample preparation	*u_sam_*	0.06
Relative combined uncertainty	*u_c_*	2.5
*U_CRM_*	*U_CRM_*	5

**Table 3:** Operating conditions of the HPLC-ICP-MS system.

ICP-MS
RF powder: 1550 W
Carrier gas: Ar 0.8 mL/min
Reaction gas: H_2_, 4 mL/min Isotope monitored: ^78^Se^+^, ^80^Se^+^ Integration time: 0.1 s (spectrum) per point Points per peak: 3
HPLC
Column: OSAKA SODA CAPCELL PAK C_18_ column
Dimensions: 250 × 4.6 mm, particle size: 5 μm
Mobile phase: 10 mmol/L sodium 1-butanesulfonic acid, 8 mmol/L tetramethylammonium hydroxide pentahydrate, 5 mmol/L malonic acid, and 5% MeOH; pH 4.0
Injection volume: 20 μL
Flow rate: 1.0 mL/min
Mode: isocratic

## Data Availability

Data is contained within this article.
